# E2F Inhibition Synergizes with Paclitaxel in Lung Cancer Cell Lines

**DOI:** 10.1371/journal.pone.0096357

**Published:** 2014-05-15

**Authors:** Courtney A. Kurtyka, Lu Chen, W. Douglas Cress

**Affiliations:** Department of Cancer Biology and Evolution, H. Lee Moffitt Cancer Center and Research Institute, Tampa, Florida, United States of America; Univesity of Texas Southwestern Medical Center at Dallas, United States of America

## Abstract

The CDK/Rb/E2F pathway is commonly disrupted in lung cancer, and thus, it is predicted that blocking the E2F pathway would have therapeutic potential. To test this hypothesis, we have examined the activity of HLM006474 (a small molecule pan-E2F inhibitor) in lung cancer cell lines as a single agent and in combination with other compounds. HLM006474 reduces the viability of both SCLC and NSCLC lines with a biological IC_50_ that varies between 15 and 75 µM, but with no significant difference between the groups. Combination of HLM006474 with cisplatin and gemcitabine demonstrate little synergy; however, HLM006474 synergizes with paclitaxel. Surprisingly, we discovered that brief treatment of cells with HLM006474 led to an increase of E2F3 protein levels (due to de-repression of these promoter sites). Since paclitaxel sensitivity has been shown to correlate with E2F3 levels, we hypothesized that HLM006474 synergy with paclitaxel may be mediated by transient induction of E2F3. To test this, H1299 cells were depleted of E2F3a and E2F3b with siRNA and treated with paclitaxel. Assays of proliferation showed that both siRNAs significantly reduced paclitaxel sensitivity, as expected. Taken together, these results suggest that HLM006474 may have efficacy in lung cancer and may be useful in combination with taxanes.

## Introduction

The retinoblastoma protein (commonly called Rb) is widely recognized as one of the most important tumor suppressors in humans. Along with the similar “pocket” proteins p107 and p130, it is responsible for regulating cell cycle progression [1]. The Rb family regulates cell cycle through binding and inhibiting the transcriptional activity of early 2 factors (E2Fs) and its tumor suppressor activity is tightly related to this function [2]. When phosphorylated (typically by CDKs 2, 4, and 6 in G1), Rb becomes inactivated, thus freeing E2Fs and allowing cell cycle progression [3]. In order to avoid aberrant cell cycle entry, CDK inhibitors such as CDKN2A (commonly known as p16) prevent CDKs from phosphorylating Rb and force cells to remain in G1 [4].

Depending upon context, E2F family members can serve as transcriptional activators that drive cell cycle progression or transcriptional repressors which restrain cell cycle progression [5]. As activators, E2Fs are recognized as being important in proliferation through their transcriptional activation of S phase genes. E2Fs activate transcription via association with histone acetyltransferase (HAT) activity [6], [7]. As repressors, E2Fs inhibit transcription of genes utilized in S phase entry by binding to their promoters and repressing transcription through their ability to bind to Rb and other pocket proteins, which in turn recruit chromatin modifiers such as histone deacetylases (HDACs) [6]–[8]. Of all the E2F family members, E2F3 is the only one individually required for cellular proliferation to occur [9]–[13]. E2F3 is important for transcription of various genes for S phase entry and has been shown to have roles in transcribing genes needed for G2/M phases (such as Aurora kinase A [14], CDC2 [15], and cyclin B1 [15], [16]). There are two E2F3 isoforms, E2F3a and E2F3b, each resulting from transcription at two different promoters. E2F3b levels remain constant throughout the cell cycle, whereas E2F3a levels fluctuate and reach peak expression levels around the G1/S transition [17]–[19]. Mouse knockout studies reveal that E2F3a and E2F3b are generally compensatory for one another [20], [21], but deletion of both isoforms is lethal [9], [20]. Finally, E2F3 is more highly expressed in multiple cancers (see [5] for a review), including lung [22] and its activity has been correlated with increased sensitivity to taxane treatment in ovarian cancers [23] and ER-negative breast cancer [24].

The CDK/Rb/E2F pathway is disrupted in virtually every instance of human lung cancer, the leading cause of cancer-related death worldwide [25]. In small cell lung cancer (SCLC), which accounts for approximately 15% of lung cancers, the most common mechanism of Rb pathway disruption is mutation of the Rb protein itself. In fact, approximately 90% of small cell lung cancers lack a functional Rb protein [26], [27]. In contrast, in non-small cell lung cancer (NSCLC), Rb mutation accounts for 15–30% of these cancers [26], [28], and deregulation of the CDK/Rb/E2F pathway more commonly occurs via silencing of the CDK inhibitor p16 [29]–[32]. In most cases of NSCLC where the *RB1* gene is intact, inhibitors of CDK4 and 6 would represent a potential way to target this pathway. This hypothesis has been examined in several clinical trials and preliminary results in breast cancer are very promising [33]–[35], suggesting that CDK/Rb/E2F pathway inhibitors may have an important role to play in the treatment of various cancers.

The benefit of CDK inhibitors may be limited to tumors in which the Rb protein remains WT and functional, and thus, reagents that could target this pathway downstream of Rb might be useful in cancers where Rb is commonly mutated (such as lung cancer). To test this hypothesis, we have examined the activity of HLM006474 against a panel of lung cancer cell lines. HLM006474 (also discussed here as 6474) is a small molecule pan-inhibitor of E2F-DNA binding [36]. Although the IC_50_ of HLM006474 is relatively high (30 µM), it has found use as a tool compound [37]–[40]. *In vivo* studies indicate that the effects of 6474 treatment on different cell lines included a reduction in cell proliferation and an increase in apoptosis and reduced invasion in a three-dimensional tissue culture model system [36]. HLM006474 may be useful in cancer prevention by leading to an increase in apoptosis and decrease of proliferation in tumorigenic human embryonic stem cells [39] as well as leading to a decrease in tumor formation in mouse embryos prone to retinoblastoma [40]. Together, these results suggest that interference with E2F activity using small molecules may have clinical application in cancer therapy. In the current work, we provide a more thorough characterization of 6474 in the context of lung cancer. HLM006474 reduces the viability of a broad array of cell lines with a biological IC_50_ that varies between 15 and 75 µM. Combination of HLM006474 with common chemotherapeutic agents cisplatin and gemcitabine demonstrates little synergy; however, HLM006474 synergizes with paclitaxel. Taken together, these results suggest that HLM006474 may have efficacy against cancers in which E2F is deregulated and may be useful in combination with other drugs that target cell cycle components.

## Materials and Methods

### Cell lines and chemical compounds

Lung cancer cell lines were obtained from ATCC or originators and were provided by the Moffitt SPORE in Lung Cancer Cell Core facility. All lines are authenticated by genotyping and maintained free of *Mycoplasma*. SCLC cell lines were grown in RPMI 1640 with 10% FBS and 1% pen/strep. NSCLC cell lines were cultured in RPMI 1640 with 10% FBS without antibiotics. HLM006474 was synthesized and validated by the Moffitt Chemistry Core as previously described [36]. Gemcitabine (Gemzar, Eli Lilly) was purchased through the Moffitt Pharmacy and dissolved in water. Cisplatin and paclitaxel were purchased from Sigma and concentrated stock solutions were prepared in dimethyl sulfoxide.

### siRNA knockdown

Cells plated at ∼50% confluency in 6-well plates were transfected using Lipofectamine 2000 (Life Technologies) according to vendor instructions with 200 pmol of either siGENOME Non-Targeting siRNA #2 (Dharmacon) or siRNA targeting E2F3a or E2F3b as described in a paper by Hurst *et al* [41]. Cells were harvested four hours after transfection for cell viability assay (as described below). The remaining cells were re-plated and harvested for Western blot analysis 24 hours after transfection.

### Viability assays

The antiproliferative activity of compounds and their combinations was evaluated using a high-throughput CellTiter-Blue cell viability assay (Promega), as previously described [42]. For the assays, 1000 cells in 24 µL were plated in 384-well plates and incubated overnight at 37°C, 5% CO_2_. The next day, the drugs were diluted in media and 6 µL of these dilutions added to appropriate wells using an automated pipetting station. Four replicate wells were used for each drug concentration. The cells were incubated with the drug for 120 hours, at which time, 5 µl CellTiter-Blue reagent (Promega Corp., Madison, WI) was added. Cell viability was assessed by the ability of the remaining treated cells to bioreduce resazurin to resorufin (579 nm Ex/584 nm Em). Fluorescence was read with a Synergy HT microplate reader (Bio-Tek Instruments, Inc., Winooski, VT). IC_50_s were determined using a sigmoidal equilibrium model regression using XLfit version 4.3.2 (ID Business Solutions Ltd.) and is defined as the concentration of drug required for a 50% reduction in growth/viability. For 3-(4,5-dimethylthiazol-2-yl)-5-(3-carboxymethoxyphenyl)-2-(4-sulfophenyl)-2H-tetrazolium (MTS) cell viability assays, CellTiter 96 AQueous One Solution from Promega was added according to vendor instructions to cells for 2 hours following treatment with drug at the noted dose for 72 hours. All experiments were performed in triplicate and repeated at least three times.

### Combination indices

The IC_50_ values obtained from single drug cell viability assays were used to design combination experiments. For 6474 combinations with cisplatin, gemcitabine and paclitaxel these ratios were 1∶1, 500∶1 and 4000∶1, respectively. For drug combination experiments, the cell viability assays were performed and the results analyzed for synergistic, additive, or antagonistic effects using the combination index (CI) method developed by Chou and Talalay [43]. Combination indices CI<1, CI = 1, and CI>1 indicate synergism, additive effects, and antagonism, respectively.

### Antibodies and western blotting

Western blots were performed as previously described [36], [44], [45]. Briefly, whole cell lysates were subjected to SDS-PAGE and transferred to PVDF membrane. Detection of proteins was accomplished using horseradish-peroxidase-conjugated secondary antibodies and enhanced chemiluminescence (ECL) purchased from Amersham or Thermo Scientific. Various antibodies used included E2F1 (C-20, sc-193, Santa Cruz), E2F3 (C-18, sc-878, Santa Cruz), PARP (#9542L, Cell Signaling Technology), and monoclonal β-actin (clone AC-15, cat no: A5441, Sigma). Adobe Photoshop CS was used to quantify Western blot band intensity readings directly from exposed films using the rectangular marquee tool/histogram and the inverted scanned film image. This same square was used for all further band readings in order to ensure that the same area was analyzed for each band. The readings were then adjusted to account for actin and background and arbitrarily normalized to the cell line H23 (assigned a value of 1).

### Real-time polymerase chain reaction

RNA was harvested from cells using the RNeasy mini kit from Qiagen. Reverse transcription polymerase chain reaction (PCR) was then performed using the iScript cDNA synthesis kit (Bio-Rad). This cDNA was then utilized in real-time PCR using either iQ SYBR Green Supermix (Bio-Rad) or PerfeCTa SYBR Green SuperMix (Quanta Biosciences, VWR). E2F1, E2F3, E2F4, tubulin, MCM2, MCM10, CCNE2, and GAPDH primers were ordered from Integrated DNA Technologies. Primer sequences are as follows: E2F1 F (5′-GCTGGACCACCTGATGAATATC-3′), E2F1 R (5′-TCTGCAATGCTACGAAGGTCCTG-3′), E2F3a/b F (5′-CGTCTCTTGGTCTGCTCAC-3′), E2F3a/b R (5′-CACTTCTGCTGCCTTGTTC-3′), E2F4 F (5′- CTGAAGAGTGTGAGTGGTC -3′), E2F4 R (5′- GCAGAGGTGGAGGTGTAG -3′), tubulin F (5′-GGGGCTGGGTAAATGGCAAA-3′), tubulin R (5′-TGGCACTGGCTCTGGGTTCG-3′), MCM2 F (5′-CTGTGTGTGGTGAGGGACAC-3′), MCM2 R (5′-CTTGTCCTGGTCCATCTGGT-3′), MCM10 F (5′-CGTCAGTGAGCAGCATGAAT-3′), MCM10 R (5′-TCCCGTTCCCATTTGTAGAG-3′), CCNE2 F (5′-CAGGTTTGGAGTGGGACAGT-3′), CCNE2 R (5′-ACTTCCTCCAGCATAGCCAA-3′), GAPDH F (5′-GAGTCAACGGATTTGGTCGT-3′), and GAPDH R (5′-TTGATTTTGGAGGGATCTCG-3′).

### Statistical analysis

For the real-time PCR analysis for the time point experiment, the difference between expression of each experimental gene (E2F1, E2F3, E2F4, CCNE2, MCM2, and MCM10) and expression of the control gene (tubulin) was calculated for each cell line at each time point. The difference between each of the non-0 hour time points and the 0 hour time point readings for each gene in each cell line was calculated using T-Tests with Welch's correction. The paclitaxel IC_50_s were log-transformed to improve normality. The correlation of E2F3 mRNA and protein expression with log paclitaxel IC_50_s was calculated using Pearson correlation coefficient. Wilcoxon rank-sum tests were used to explore the difference of cell viability in control siRNA treatment with either E2F3a or E2F3b siRNA treatment.

## Results

### HLM006474 has broad antiproliferative activity

To examine the potential of 6474 in the treatment of lung cancer, we determined the 6474 IC_50_ in seventeen lung cancer cell lines (**Table 1**). This analysis included eight non-small cell lung cancer lines (NSCLC) and nine small cell lung cancer lines (SCLC). In this viability assay, cells were plated at low density on day zero and were grown in the presence of various 6474 concentrations for five days. After five days, the relative viability of cells was determined by staining with CT-Blue (Promega). The results reveal that the 6474 IC_50_ ranges from ∼15 to ∼75 µM across the seventeen cell lines. The average biological IC_50_ of all the cells lines was 31.4 (+6.1) µM, which is essentially identical to the biochemical IC_50_ of 29.8 (±7.6 µM, previously reported [36]. There was no statistically significant difference between SCLC and NSCLC.

**Table 1:** HLM006474 IC_50_ values across various cell types.

Cell Line	Tumor Type	IC_50_	STDEV
A549	NSCLC	31.80	12.90
NCI-H1299	NSCLC	27.30	16.50
NCI-H1650	NSCLC	34.00	3.60
NCI-H1975	NSCLC	44.30	12.10
NCI-H292	NSCLC	28.90	3.10
NCI-H358	NSCLC	19.10	4.60
NCI-H441	NSCLC	15.50	3.40
NCI-H661	NSCLC	23.00	3.20
DMS-79	SCLC	22.30	3.10
SCLC-16HC	SCLC	24.90	4.00
SCLC-16HV	SCLC	51.40	10.90
SCLC-86M1	SCLC	15.70	2.40
DMS114	SCLC	23.80	1.50
NCI-H209	SCLC	21.90	7.19
NCI-H69	SCLC	53.70	5.44
NCI-H82	SCLC	21.30	3.02
NCI-N417	SCLC	75.10	6.96
	*NSCLC Ave.*	*27.99*	*7.43*
	*SCLC Aver.*	*34.46*	*4.95*
	*Overall Aver.*	*31.41*	*6.11*

The IC_50_ of various cell lines was determined as described in the methods. STDEV refers to the standard deviation of indicated values calculated using Excel STDEV function. The average IC_50_s of the NSCLC and SCLC groups are calculated in bold along with STDEVs. The differences are not statistically significant.

### Short treatments with HLM006474 lead to increased expression of several known E2F-regulated genes

**Figure 1:**
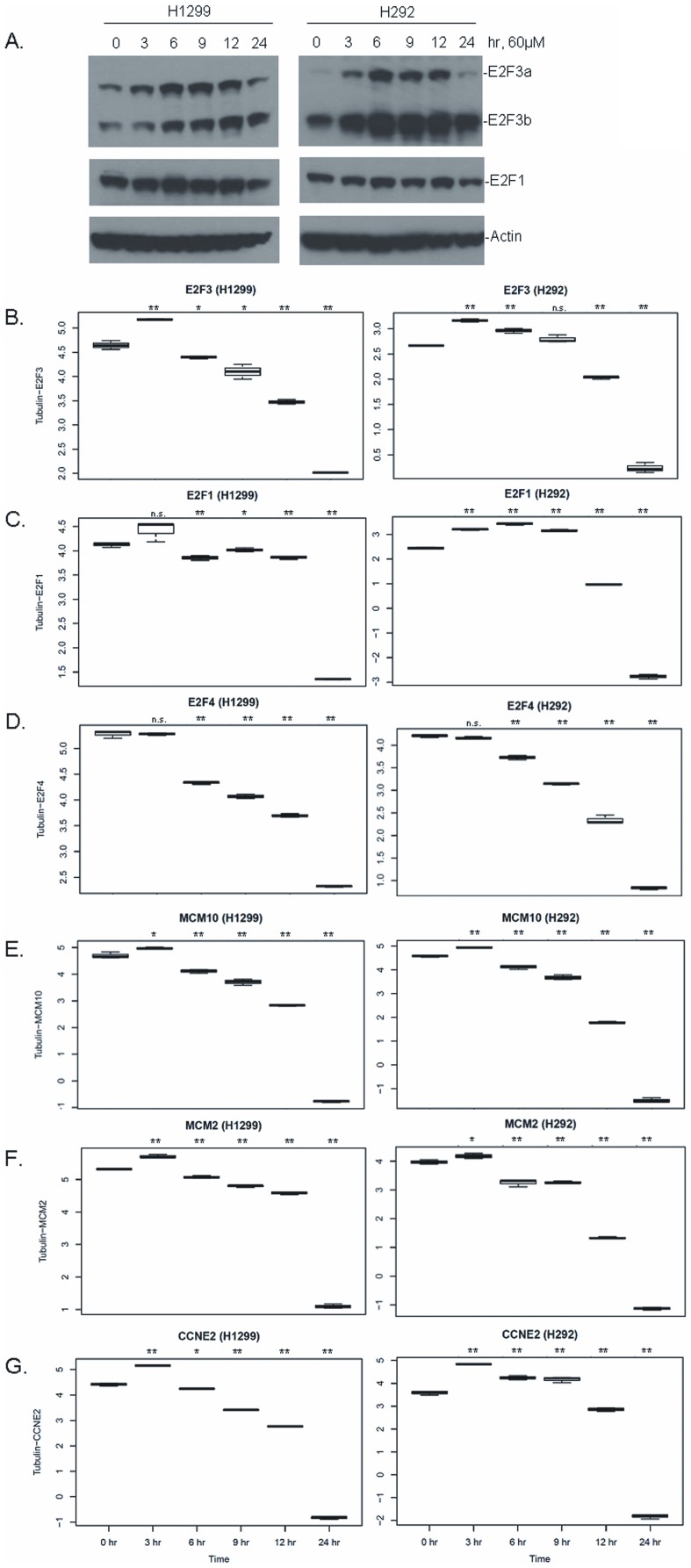
Expression of common E2F-regulated genes increases in early time point treatments with HLM006474. *A*. The NSCLC cell lines H1299 and H292 were treated with 60 µM HLM006474 for 0, 3, 6, 9, 12, and 24 hours, then harvested and examined through Western blots. Modest increases in E2F1 and more dramatic increases in E2F3a and b protein levels were noted at early time point in both cell lines. *B–D*. mRNA was harvested from cells that were treated as described in 1A, converted to cDNA using RT-PCR, and analyzed for expression levels of E3F3 (*B*), E2F1 (*C*), and E2F4 (*D*) using real-time PCR and tubulin as a control. Short treatments with 6474 altered mRNA expression levels of E2F3 and E2F1, but not E2F4. *E–G.* mRNA expression of well-known genes commonly regulated by E2Fs were analyzed in a manner similar to the previous description in 1B–D. Expression levels of MCM10 (*E*), MCM2 (*F*), and CCNE2 (*G*) mRNA were analyzed with tubulin as a control and were all noted to be more highly expressed following short treatments with 6474. Notes: n.s. represents not significant, * represents p<0.05, ** represents p<0.01.

As a component of our analysis of HLM006474, we examined the expression of E2F family members following treatment by Western blotting. NSCLC cell lines H292 and H1299 were treated with 60 µM HLM006474 for 0, 3, 6, 9, 12, and 24 hours and analyzed via Western blot (**Figure 1** **A**). Surprisingly, protein levels of both E2F3a and b isoforms increased in early time points (typically around 6–9 hours). Levels of the E2F1 protein increased more modestly following treatment, peaking between 6 and 12 hours and returning to baseline levels after 24 hours. In real-time PCR analysis with tubulin as a control, E2F3 mRNA levels increased significantly after 3 hours of treatment and then decreased in each subsequent time point (**Figure 1B**), while E2F1 mRNA expression levels were significantly increased after short treatment times in H292 alone (**Figure 1C**) and E2F4 levels remained constant or decreased at each time point (**Figure 1D**). It was also noted that some genes commonly regulated by E2Fs; MCM10 (**Figure 1E**), MCM2 (**Figure 1F**), and CCNE2 (**Figure 1G**); were more highly expressed in early time points in a manner comparable to the changes seen in E2F3 mRNA expression. The results shown in **Figure 1** suggest that treatment with an inhibitor of E2F-DNA binding can result in the activation of E2F-regulated targets, including auto-regulated E2F family members. The E2F family is known to actively repress transcription [46]–[49], and thus, we propose that treatment with HLM006474 may displace E2F-repressive complexes and thereby activate transcription of E2F-regulate genes that are predominantly repressed by E2F complexes. To explore this possibility, we used siRNA specifically against E2F1, E2F3a, E2F3b, E2F4 and Rb to deplete two NSCLC cell lines of various E2F complexes. Microarray was performed to examine the effect of these siRNAs on the expression of an E2F signature previously defined based on E2F3 overexpression [50]. The results, which will be published elsewhere, demonstrate that depletion of individual E2Fs results in many E2F signature genes being activated, as would be expected from a de-repression model.

### HLM006474 synergizes with paclitaxel

**Figure 2:**
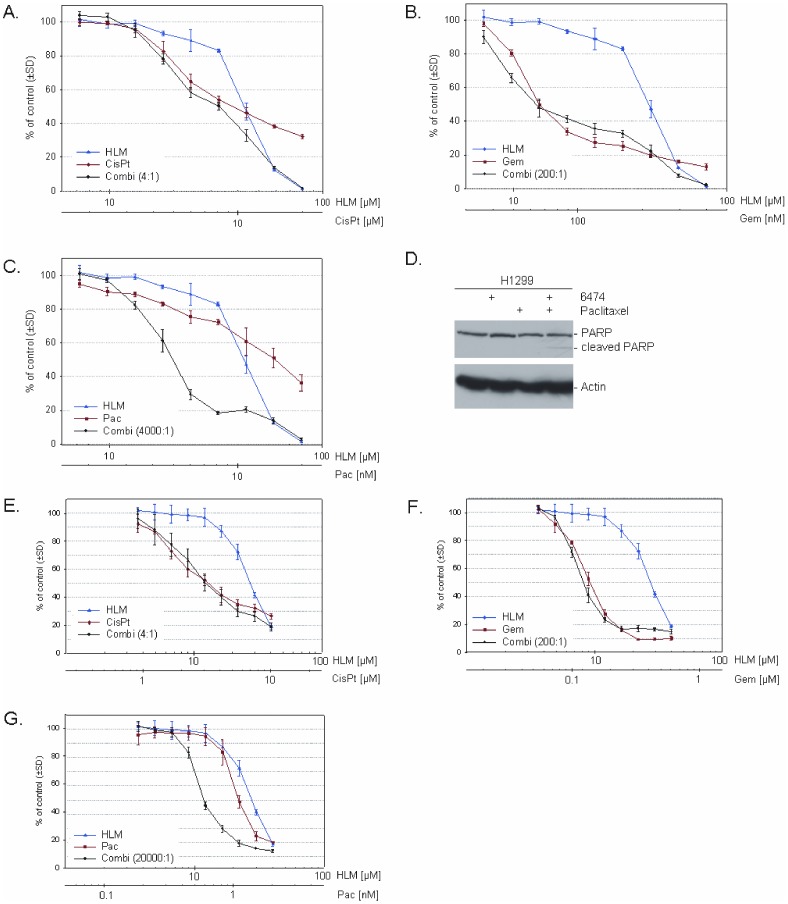
HLM006474 synergizes with paclitaxel, but not with cisplatin or gemcitabine. *A–C.* H1299 cells were subjected to viability assays in the presence of 6474 (HLM) combined with cisplatin (*A*, CisPt), gemcitabine (*B*, Gem) and paclitaxel (*C*, Pac), as indicated (see methods). Results reveal synergy with paclitaxel (CI average 0.98) and antagonism with cisplatin and gemcitabine (CI average 1.40 and 1.39, respectively). *D*. Western blotting reveals that in H1299 cells treated for 72 hours with 20 µM 6474 alone, 5 nM paclitaxel alone, or the combination of the two, 6474 and paclitaxel synergize in the induction of PARP cleavage. *E*–*G*. H292 cells were tested in similar viability assays to determine the efficacy of 6474 (HLM) combined with cisplatin (*E*, CisPt), gemcitabine (*F*, Gem) and paclitaxel (*G*, Pac). As seen in H1299 cells, 6474 synergizes with paclitaxel (CI average 0.96), but is antagonistic with cisplatin (CI average 1.51) and gemcitabine (CI average 1.46).

Having established that 6474 has anti-proliferative effects on lung cancer cell lines, but may influence E2F-regulated genes in a complex manner, we sought to determine if 6474 would synergize with chemotherapeutic drugs commonly used in lung cancer treatment. H1299 cells were treated with 6474 alone and in combination with cisplatin, gemcitabine and paclitaxel. Combinations were chosen based on the IC_50_ of the cells to the individual compounds and combination indices were calculated [43]. **Figure 2** reveals that there is antagonism between 6474 and cisplatin (panel **2A,** CI average 1.40) and gemcitabine (panel **2B**, CI average 1.39). In contrast, 6474 weakly synergizes with paclitaxel (panel **2C**, CI average 0.98). To further explore the nature of the synergy between 6474 and paclitaxel, H1299 cells were treated with modest doses of each drug alone or in combination and utilized in Western blot analysis. The appearance of cleaved PARP in cells treated with the drug combination confirms that the combination of 6474 and paclitaxel induces more apoptosis than either drug alone (**Figure 2D**). To explore whether this synergy would be observed in additional cell lines, we also examined the NSCLC cell line H292. As in the case of H1299 cells, 6474 synergized with paclitaxel (panel **2G**, CI average 0.96), but showed no synergy with cisplatin (panel **2E**, CI average 1.51) or gemcitabine (panel **2F**, CI average 1.46).

### E2F3 levels impact sensitivity to paclitaxel

**Figure 3:**
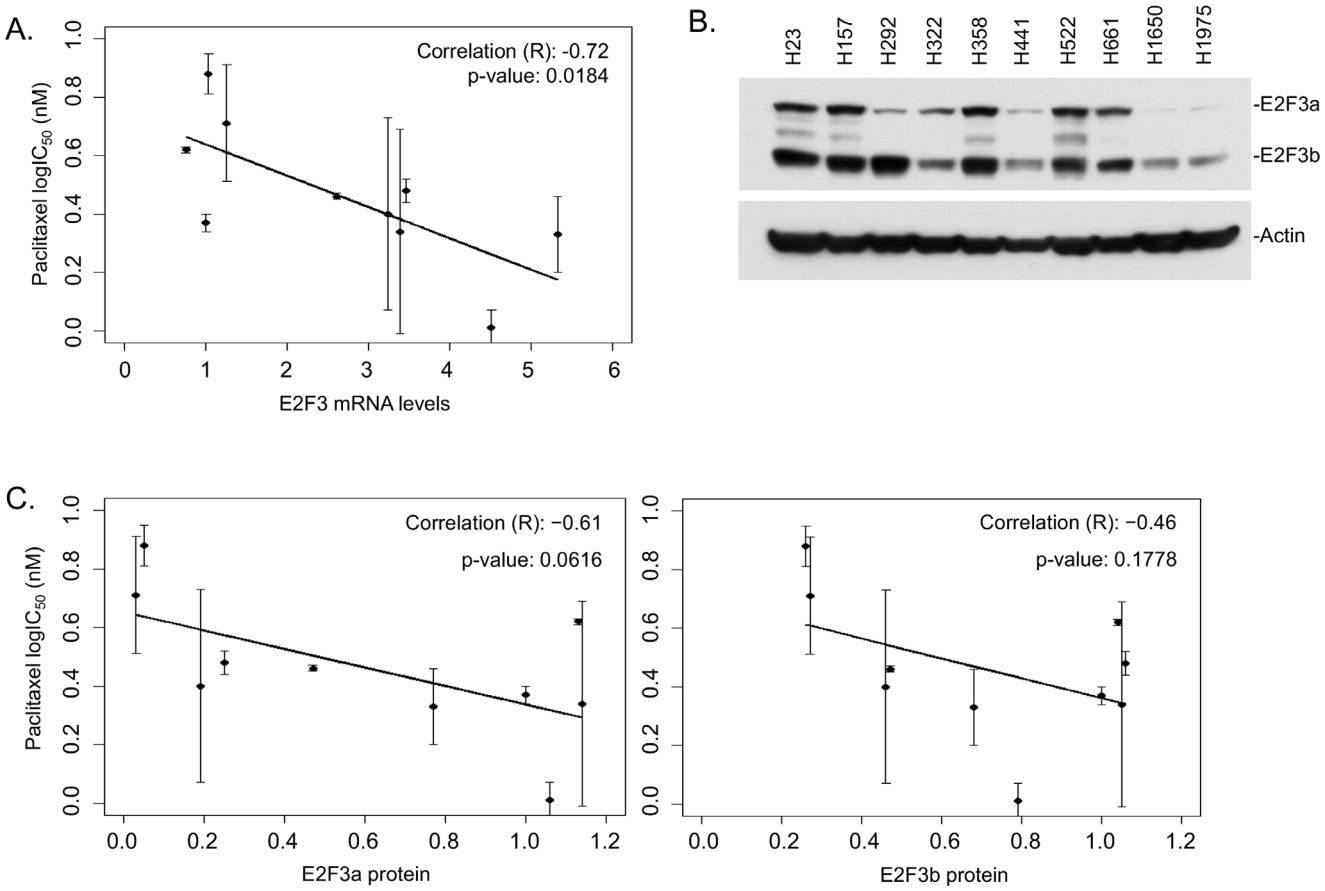
Sensitivity to paclitaxel correlates with E2F3 levels in NSCLC. *A*. cDNA from ten NSCLC cell lines were utilized in real-time PCR to detect endogenous E2F3 expression levels (as compared to GAPDH as a control). The expression levels were then compared to the paclitaxel logIC_50_ of each line and graphed as shown. *B*. Lysates from ten NSCLC cell lines were prepared and ran in a Western blot to detect endogenous E2F3 levels. *C*. Densitometrically analyzed values of the protein expression levels (as compared to β-actin as a control) were then graphed against the paclitaxel logIC_50_ for each line as shown.

**Figure 4:**
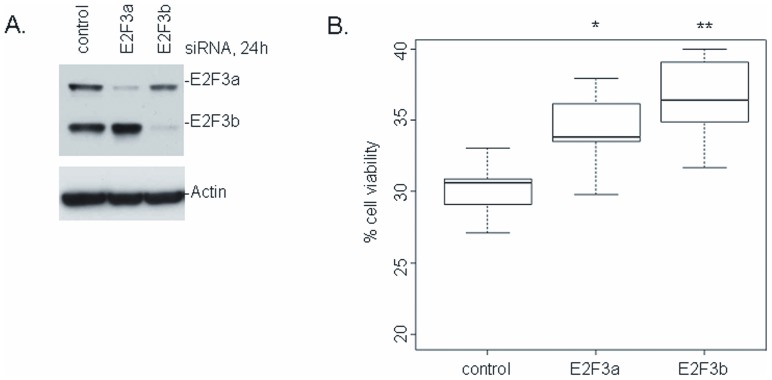
Depletion of E2F3 alters sensitivity to paclitaxel. *A*. H1299 cells were transiently transfected with 200 pmol control, E2F3a, or E2F3b siRNA and harvested after 24 hours. Western blots of these lysates demonstrate the extent of E2F knockdown. *B*. MTS assays were used to determine the sensitivity of each cell line to 50 nM paclitaxel. Cells with diminished E2F3 levels were more viable in the presence of paclitaxel than control cells. Notes: * represents p<0.05, ** represents p<0.01.

The observations that an E2F inhibitor could synergize with paclitaxel and the previously discussed increase in E2F3 levels following early time point treatments with HLM006474 suggested that E2F3 activity might play a role in paclitaxel sensitivity. Real-time PCR was used to analyze the endogenous expression of E2F3 (with GAPDH serving as an internal control), and then these values were correlated to the paclitaxel logIC_50_ of each cell line (**Figure 3A**). Lysates from ten NSCLC cell lines were run in Western blots (**Figure 3B**) and densitometrically analyzed using β-actin as a control. These E2F levels were then plotted against the corresponding paclitaxel logIC_50_ values (**Figure 3C**). In both the real-time PCR and Western blot analyses, a strong inverse correlation between E2F3 and paclitaxel IC_50_ was noted. To more formally test this hypothesis, we used siRNA to deplete H1299 cells of E2F3a and E2F3b and then determined their sensitivity to paclitaxel as measured in MTS assays. Western blotting reveals an almost complete knockdown of the targeted E2Fs in the H1299 cells (**Fig 4A**). Control siRNAs did not affect E2F expression. As expected, **Fig 4B** reveals that the cells with decreased E2F3 levels were less sensitive to paclitaxel.

## Conclusions

The CDK/Rb/E2F pathway represents a good target for the treatment of various solid tumors. Although development has been slow due to toxicity of early compounds, CDK inhibitors are starting to gain traction in clinical trials [33], [51], [52]. We propose that targeting the CDK/Rb/E2F pathway even further downstream, at the E2F level, may also be of value. Thus, we have examined the potential of a pan-E2F inhibitor, HLM006474, in the treatment of lung cancer.

**Figure 5:**
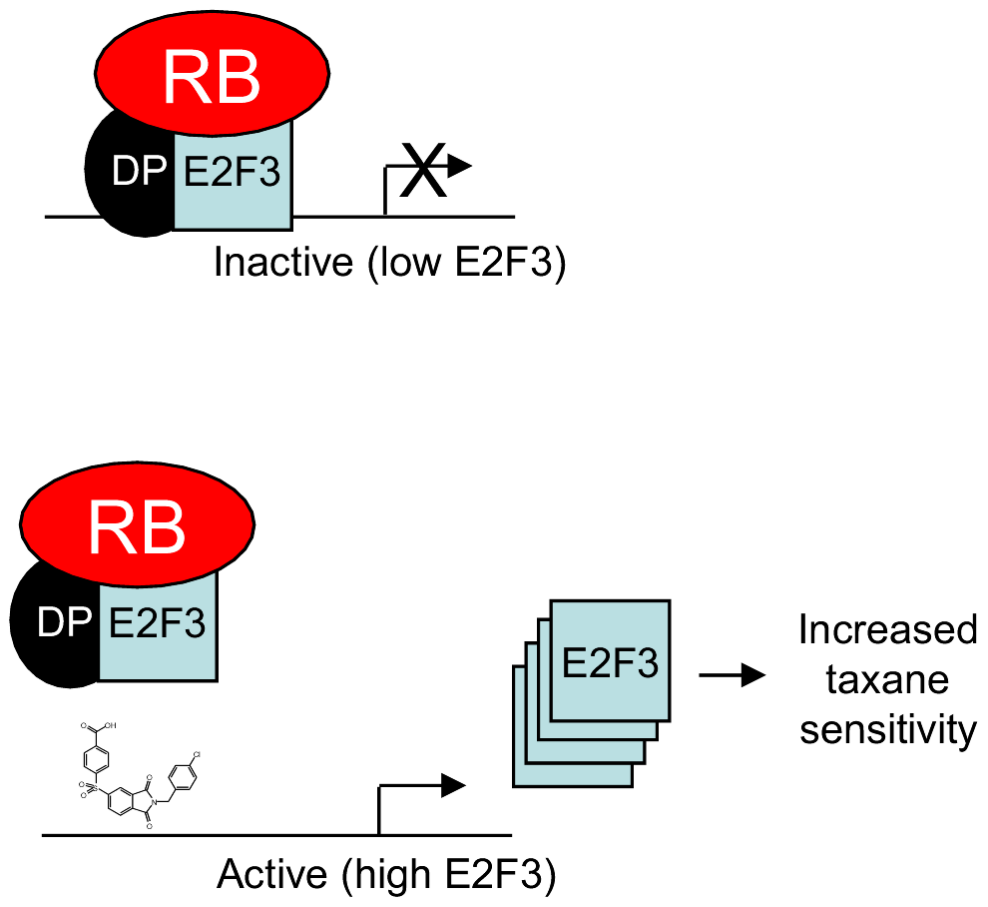
Model of HLM006474 synergy with paclitaxel. In untreated cells, E2F/dimerization partner (DP)/Rb complexes predominate, and thus, E2F3 levels are relative low (as well as many other E2F-regulated genes). Treatment with HLM006474 disrupts the E2F/DP/Rb repressive complexes from binding DNA, resulting in increased transcription of E2F3 (as well as many other E2F-regulated genes). In this model, the elevated levels of E2F3 sensitize cells to taxane treatment, as previously demonstrated, through an unknown mechanism.

We propose the model in **Figure 5** to explain our results. First, we observe that treatment with 6474 leads to a transient increase in not only E2F3 protein and mRNA expression levels, but also an increase in many other E2F-regulated transcripts. These counter-intuitive observations are reasonable based on the long-known observation that E2Fs are active repressors of transcription [46]–[49]; however, they do raise concerns that pan-E2F complex inhibition may have unwanted consequences. Thus, future E2F-targeted compounds should selectively block transcription activating E2F complexes and spare transcriptional repressing E2F complexes. Second, we believe the increased levels of E2F3 (and likely other E2F-regulated genes) increase the sensitivity of the cells to paclitaxel. We base this conclusion on the correlation observed between normal E2F3 levels and paclitaxel sensitivity, as well as the results of E2F3 siRNA experiments. This documents the first link between levels of E2F3 and paclitaxel in NSCLC, though a relationship between high levels of E2F3 activity and increased sensitivity to paclitaxel has been previously observed in ovarian [23] and ER-negative breast cancer [24]. The mechanism by which E2F3 generates sensitivity to paclitaxel is unknown. One possibility is that it relates directly to E2F3's role in cell cycle. For example, in cells with high E2F3 levels, it would be expected that the cells would proliferate more, thus giving cells a greater opportunity to enter M phase (where paclitaxel would be most effective). However, this explanation alone would suggest that these cells should be more sensitive to gemcitabine as well due to entering S phase more often. It might be more likely that greater sensitivity to paclitaxel is due to apoptosis-regulating genes becoming highly expressed due to the increase in E2F3. Also, it has been previously noted that overexpression of E2F3 leads to an enrichment of genes that are microtubule-related [23], so this could perhaps explain the correlations we see between E2F3 levels and paclitaxel sensitivity. Likewise, as mentioned previously, E2F3 has been noted to have a role at the G2/M checkpoint through its regulation of expression of Aurora kinase A [14], CDC2 [15], and cyclin B1 [15], [16], which may also point to higher levels of E2F3 leading to an increase in cells entering that phase of the cell cycle and perhaps then increasing paclitaxel sensitivity.

We have demonstrated that HLM006474 is effective in lung cancer cell lines. Furthermore, we have shown that 6474 synergizes well with paclitaxel, potentially due to 6474's effects on E2F3 levels. Taken together, these results suggest that potent, specific activator E2F inhibition may be useful in the treatment of NSCLC in the future (especially in combination with other agents), and that E2F3 levels may be a good predictor of paclitaxel sensitivity in NSCLC.
